# Theory-Driven Process Evaluation of the SHINE Trial Using a Program Impact Pathway Approach

**DOI:** 10.1093/cid/civ716

**Published:** 2015-11-11

**Authors:** Mduduzi N. N. Mbuya, Andrew D. Jones, Robert Ntozini, Jean H. Humphrey, Lawrence H. Moulton, Rebecca J. Stoltzfus, John A. Maluccio

**Affiliations:** 1 Zvitambo Institute for Maternal and Child Health Research, Harare, Zimbabwe; 2 Division of Nutritional Sciences, Cornell University, Ithaca, New York; 3 Department of International Health, The Johns Hopkins Bloomberg School of Public Health, Baltimore, Maryland; 4 School of Public Health, University of Michigan, Ann Arbor; 5 Department of Economics, Middlebury College, Vermont

**Keywords:** process evaluation, program impact pathway, intention to treat, per protocol

## Abstract

Two reasons for the lack of success of programs or interventions are poor alignment of interventions with the causes of the problem targeted by the intervention, leading to poor efficacy (theory failure), and failure to implement interventions as designed (program failure). These failures are important for both public health programs and randomized trials. In the Sanitation Hygiene and Infant Nutrition Efficacy (SHINE) Trial, we utilize the program impact pathway (PIP) approach to track intervention implementation and behavior uptake. In this article, we present the SHINE PIP including definitions and measurements of key mediating domains, and discuss the implications of this approach for randomized trials. Operationally, the PIP can be used for monitoring and strengthening intervention delivery, facilitating course-correction at various stages of implementation. Analytically, the PIP can facilitate a richer understanding of the mediating and modifying determinants of intervention impact than would be possible from an intention-to-treat analysis alone.

The emergence of implementation science over the past decade has advanced understanding of the many possible impediments to delivery and utilization that can limit the potential impacts of proven health and nutrition interventions [1–3]. One area in which its added value is most apparent is through embedding theory-driven process evaluation directly into evaluation studies, including randomized trials [4, 5]. This typically entails conceptually articulating how an intervention has been designed to work (ie, the program theory) and elucidating and measuring intermediate outcomes that need to be achieved for it to work as intended [6–8]. Such theory-driven approaches can provide generalizable knowledge and explain positive, modest, and insignificant results in a single intervention study. Moreover, they can help to identify the intervention components most responsible for the observed effects [9, 10], as well as inform the scale-up of efficacious interventions and programs [3, 11, 12].

Within the growing body of literature in this area [13–20], 2 published examples in particular illustrate the value of theory-driven process evaluation in nutrition research. The first is a cluster-randomized trial (CRT) of an educational intervention in Peru, demonstrated to have had a positive impact on both ponderal and linear growth [21]. Consistent with the linkages laid out in the a priori program theory and therefore strengthening the plausibility of the results, the authors demonstrated that better health center implementation of the intervention positively influenced caregiver exposure, which was in turn positively associated with caregiver message recall, which was in turn positively associated with key feeding behaviors [22]. Furthermore, the trial was found to have significant impacts despite less than complete implementation—intervention components were delivered at 50%–90% of expectations, while fidelity of implementation or adherence to the intervention protocol was only 28%–70% [23]. This raises the possibility that even larger effects might be possible if better implementation could be achieved. In the second illustrative example, no differential growth effect was observed in a multisite (Guatemala, Pakistan, Zambia, and Democratic Republic of the Congo) trial of 2 child feeding interventions (daily meat intake compared with an equicaloric micronutrient fortified cereal) in contexts with prevalent stunting [24]. A theory-driven process evaluation confirmed that there were no differences in visits, deliveries of the study foods, message recall, or rates of consumption of study foods between the treatment groups [25]. This process evaluation, and high fidelity of implementation observed, increased confidence that the trial's null finding was not due to differences or inconsistencies in protocol implementation. Additionally, message recall was associated with linear growth velocity irrespective of treatment group. This underscored the importance of the study messages, suggesting that targeted infant feeding education for low-literacy populations, for whom message recall was lower, could be efficacious [25].

The Sanitation Hygiene Infant Nutrition Efficacy (SHINE) Trial is designed to test the independent and combined effects of 2 village health worker (VHW)–delivered intervention packages to improve water, sanitation, and hygiene (WASH) and infant feeding behaviors with the aim of improving length and hemoglobin concentration (or reducing stunting and anemia respectively) at 18 months of age. The design is a cluster-randomized, 2-factorial, community-based intervention trial in 2 rural districts of Zimbabwe. Whereas the factorial design allows for efficient comparison of 2 interventions with hypothesized independent and additive effects, the cluster-randomized design is necessitated by the theoretical (eg, health behavior is in part socially constructed) and practical (eg, cost and implementation feasibility) considerations of delivering sustained behavior change communication to rural communities [26]. The unit of randomization is a group of households within the (geographically contiguous) catchment area of 1–4 VHWs.

The overall approach of the SHINE Trial is to implement a health system-based project in which randomized behavior change interventions are delivered by VHWs. In SHINE, VHWs are dually supervised; (1) by Ministry of Health and Child Care nurses in carrying out their regular duties, and (2) by SHINE research staff in carrying out the additional tasks required by the trial. VHWs are trained to visit participants monthly, deliver messages in accordance with behavior change intervention modules relevant to their randomized arm, and deliver corresponding inputs such as soap, water chlorination agents, and small-quantity lipid-based nutrient supplements (LNSs). As such, the SHINE Trial is a complex intervention within a complex system [27–29]. Evaluating and understanding the impact of such an intervention is challenging because the pathways to impact are multiple and subject to effect modification [8].

Intention-to-treat (ITT) analysis of the main outcomes is the standard approach for statistical tests of the hypothesis of null effects of treatment in a randomized trial [30] and is the primary analysis approach of the SHINE Trial [31]. The ITT estimates the effect of treatment as randomly allocated, which may substantially differ from the treatment effect for those who actually both received and took up the intervention [30, 32]. As such, unless implementation and uptake are perfect, ITT estimates do not directly reveal whether it was the improved sanitation/hygiene and infant nutrition practices themselves that have led to any observed changes in length (stunting) and hemoglobin concentration (anemia).

In this article, we present the theory-driven process evaluation embedded within the SHINE Trial. First, we present the SHINE program impact pathway (PIP), which details how we hypothesize the interventions will achieve their desired effects. Second, we describe how we are measuring the key mediating domains at each step in the PIP. Third, we describe how we use the PIP operationally. Finally, we discuss its application in 2 broad analytic approaches: a PIP analysis that examines the flow of the intervention impact at each step along the hypothesized pathway and per-protocol analyses that explore treatment effects among the treated at each step.

## THE PIP LINKING WASH AND INFANT FEEDING WITH STUNTING AND ANEMIA

**Figure 1.**
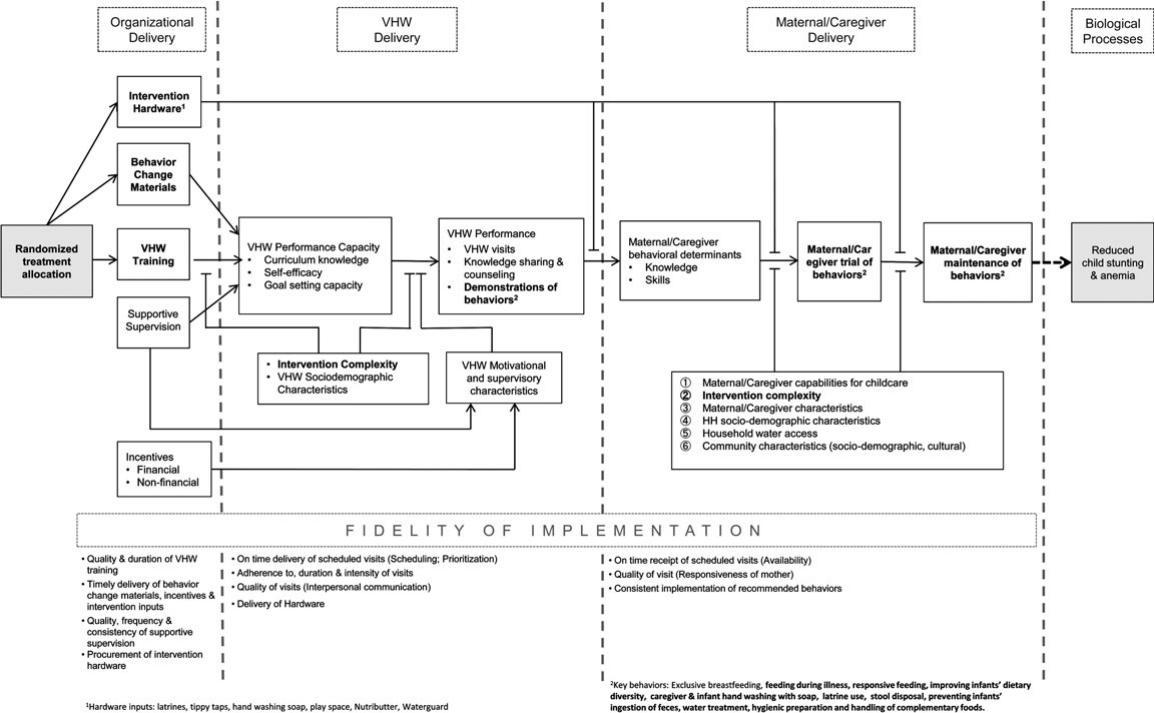
Program impact pathway linking village health worker (VHW) treatment allocation with stunting and anemia outcomes. Boldface text denotes those constructs that differ in accordance with the randomized design.

As with a CRT of any complex intervention, the SHINE Trial cannot randomize the *actual behaviors* carried out at the level of the unit of randomization (communities served by 1–4 VHWs), let alone at the individual level. Rather, SHINE randomly allocates VHWs to receive training for, offer, and deliver, 1 of the 4 treatments; and under black box [33] ITT assumptions, this VHW allocation is what leads to improvements in length and hemoglobin concentration. The PIP, by detailing the intervening steps illustrated in Figure 1, postulates a theory of what happens inside that black box.

In collaboration with the Ministry of Health and Child Care, SHINE staff train VHWs and provide behavior change materials, intervention hardware, and supervisory support (including financial and nonfinancial incentives) to effect 4 sequential intermediate processes: (1) improved VHW performance capacity, as measured by their relevant knowledge, self-efficacy, and goal-setting capacity; (2) actual VHW performance as measured by the degree to which it conforms with intervention design (15 SHINE visits to all participating households, delivery of behavior change interventions); (3) improved maternal and household behavioral determinants, as measured by their relevant knowledge, message recall, and attitudes toward recommended behaviors; and (4) improved trial and maintenance of WASH and/or infant feeding behaviors by the study participants, as measured by the evidence of recommended behaviors being practiced. These improved behaviors represent proximate outcomes to stunting and anemia that directly precede the biologic processes described in Prendergast et al [34].

Recognizing that effect heterogeneity is inevitable in the program pathway of a complex intervention [35], the PIP posits several primary effect modifiers along the pathway of impact. First, individual VHW characteristics (sociodemographic characteristics such as age, sex, and marital status; motivational and supervisory characteristics) can influence performance capacity as well as the translation of this capacity into actual performance. Second, maternal and household characteristics are likely to modify trial and maintenance of recommended practices. Based on emerging research on the potentially important role of skills and attributes of a caregiver that determine his or her ability to provide appropriate care for a young child [36, 37], we were particularly careful to collect information on a range of such attributes, which we have termed caregiver capabilities [38]. These include social support for mothering, control of household resources, mental health, time availability or lack of perceived time stress, perceived physical health, and mothering self-efficacy. Also, baseline sociodemographic characteristics of the households (eg, socioeconomic status, education, food security, water access) are likely to modify trial and maintenance of sanitation/hygiene and infant nutrition behaviors and therefore the overall trial's intervention impact. Third, VHW performance and also maternal and household adoption of new behaviors might be differentially affected when the 2 interventions (sanitation/hygiene and infant nutrition) are combined, compared with when they are delivered separately in our factorial design (ie, the effect of intervention complexity) [39].

## USE OF THE PIP TO MONITOR FIDELITY OF IMPLEMENTATION

One way in which we operationalize the PIP is by monitoring fidelity of implementation (FOI; the degree to which interventions are implemented as intended). In SHINE, FOI is operationalized to include the delivery process (how regularly and timely intervention commodities [latrine, soap, LNS] are delivered, and what VHWs do), the quality of delivery (how well they do it), and participant responsiveness (how households respond) [40].

Ensuring high treatment fidelity in a randomized trial of a complex intervention is important for ethical, as well as statistical, reasons. It is crucial for ensuring internal validity (ie, fair comparison of treatments) and generalizability of results [41]. Internal validity is threatened by differences between planned and actual administration of treatments. Generalizability is enhanced when it is well understood which components were or were not fully implemented. Furthermore, heterogeneity within or between delivery agents (eg, VHWs) can inflate error variance and decrease statistical power [41]. As such, it is necessary for trials to document and report fidelity assessments over time and across space, and to use them for program monitoring and improvement.

Process evaluation is the principal approach for identifying discrepancies between the program as intended and the program as implemented [42]. The SHINE Trial PIP articulates the intended pathways and processes including organizational actions, delivery agent capabilities and performance, behavior change interactions, and trial and adoption of target practices. Operationally, the study team uses the PIP for to strengthen programming, and facilitate ongoing course correction of implementation. For example, the following questions were addressed in several process examinations undertaken by the study team during the multiyear implementation process.

- Are VHWs receiving regular (monthly) and systematic (record review, structured observation, troubleshooting) on-the-job support and feedback from their supervisors?
- Are VHWs delivering the required number of behavior change messages at the required times with the required material inputs and correct education techniques?
- Are VHWs motivated and satisfied with their remuneration, supervision, and provision of tools of the trade? Do they feel adequately equipped to do their work, and do they feel valued, respected, and well supervised?

Answers to these questions represent actionable information that can be used to address implementation problems on an ongoing basis. For example, reported dissatisfaction with monetary allowances identified during the baseline round of VHW interviews informed the formulation of a performance-based incentive package begun in December 2013 to improve VHW motivation and job performance outcomes [43].

## METHODS AND MEASUREMENTS

We collect process evaluation data through record reviews, structured observations, interviews with VHWs and interviews with study participants at outcome measurement time points [31]. All enumerators, research nurses, and supervisors were trained and standardized on the various methods.

### Record Reviews

VHWs maintain a module-delivery schedule for each mother they recruit into SHINE. These schedules specify which module should be delivered when, as well as allowable and acceptable windows around the target date. VHWs record when the module was delivered. VHWs also maintain registers in which they record their activities, such as prospective pregnancy surveillance. Supervisors routinely inspect this documentation and collect these data from each VHW during their scheduled monthly supervisory contacts. These data will be used to characterize supportive supervision (frequency of VHW-supervisor contacts) and VHW performance.

### Structured Observations

VHW supervisors conduct structured observations of all VHWs to assess and document VHW interactions with study participants and adherence to behavior change intervention protocols. For each VHW, these observations are conducted during the first delivery of each new behavior change intervention module and quarterly thereafter. The assessment tools consist of Likert-type, multiple-choice, dichotomous, and subjective qualitative items that are used to assess specific behaviors of VHWs. Measures of VHW performance, such as lesson delivery scores, will be derived from these data.

### Interviews With VHWs

Research staff (part-time enumerators) administer a questionnaire to each VHW (following their informed consent as a research subject) 3 times, at baseline, midline, and endline. Data on sociodemographic, supervisory and motivational characteristics [14], curriculum knowledge, [18] and goal-setting capacity are collected.

### Interviews With Participants

Research nurses administer questionnaires to participating women during 2 antenatal and 5 postnatal visits between recruitment at approximately 14 weeks of gestation and 18 months postpartum. Data collected include sociodemographic information, exposure to behavior change interventions, curriculum knowledge, maternal capabilities for caregiving [38], and WASH and infant feeding behaviors. A questionnaire module ascertains different indicators of household water access: source, type, walking time [44], distance of water for drinking and water for uses other than drinking, and 24-hour recall of household water collection. A composite measure of knowledge-sharing efficacy [18] will be derived from combining data on the curriculum knowledge of participating women with curriculum knowledge of VHWs, to assess VHW performance in knowledge sharing. Also, we will explore the computation of separate WASH and infant feeding behavior scores incorporating the behaviors promoted by the SHINE interventions. Relative socioeconomic (wealth) status will be derived using a principal components analysis that includes data on household assets, income, expenditures, and access to agricultural land at the time of the baseline household visit.

A summary of the data collected, data sources, the indicators derived, and timing of data collection is presented in Supplementary Appendix Table 1.

### Use and Implications of PIP for Statistical Analysis

The full PIP, from randomized treatment allocation to reduced childhood stunting and anemia, elucidates several intermediate steps, a number of potential modifiers at each step, and different potential measures to characterize each step (including of FOI at delivery/receipt steps such as between the VHW and caregiver or between the caregiver and infant). Above and elsewhere [31], we describe our efforts to collect data that characterize this complex system. However, without making a large number of assumptions, it is infeasible to model this full PIP in a single statistical analysis. Instead, we will carry out a series of separate “partial” analyses that, when taken as a whole, test the theorized links in the PIP [7]. The statistical approaches we use complement the analysis plan for the primary outcomes of the trial [31], applied to the intermediate outcomes in the PIP.

More specifically, we will conduct analyses of intermediate outcomes at each step along the PIP: (1) VHW performance capacity; (2) VHW performance; (3) maternal behavioral determinants/capacity; and (4) maternal behavior/performance. We will employ 2 analytical approaches in these analyses: (1) ITT based on the original randomized design and examining each intermediate outcome separately as an endpoint; and (2) per-protocol analyses linking together intermediate steps and conditional on specific prior outcomes or achievements in an earlier step, such as high FOI. For both approaches we will, via interactions, explore the role of pre-specified modifiers. Examples and potential hypotheses to be explored are presented in Table 1.

**Table 1.** Potential Program Impact Pathway Hypotheses and Their Estimation Strategies

PIP Domain(s)	Hypothesis	Primary Estimation Strategy
VHW performance capacity	The performance capacity (curriculum knowledge, self-efficacy and goal-setting capacity) of VHWs will differ by their sociodemographic characteristics (eg, age, sex, tenure).	Intention-to-treat
VHW performance, FOI-VHW delivery	The fidelity of intervention delivery among VHWs assigned to implement both the WASH and IYCF interventions will be lower than the fidelity of intervention delivery among those VHWs assigned to implement only the WASH or IYCF interventions	Intention-to-treat
VHW: performance Capacity → performance	VHWs with greater performance capacity will deliver the interventions with higher fidelity; this process will be modified by individual VHW characteristics.	Per protocol
Caregiver trial and maintenance of behaviors	For the WASH intervention, mothers in households with greater access to water will actively practice hand washing with soap to a greater extent than households with less access to water.	Intention-to-treat
Caregiver trial and maintenance of behaviors	For the infant nutrition intervention, more food-secure households will take up IYCF behaviors to a greater extent than less food-secure households.	Intention-to-treat
FOI-VHW delivery → caregiver behavioral determinants	Mothers who receive interventions delivered at higher fidelity will attain more knowledge and skills; this process will be modified by individual VHW characteristics, maternal capabilities, household wealth, and the relationship between the VHW and mother.	Per protocol
Caregiver: behavioral determinants → trial and maintenance of behaviors	Mothers who attain more knowledge and skills will more fully implement the promoted behaviors; this process will be modified by maternal capabilities for childcare and the socioeconomic status of her household.	Per protocol
Caregiver maintenance of behaviors	Among all participants, we hypothesize that wealthier households and households with more highly educated mothers will take up intervention behaviors to a greater extent than less wealthy households and households with less educated mothers, respectively.	Intention-to-treat
FOI-VHW delivery → length and hemoglobin concentration	Children of participants who received the treatments as intended will have higher length-for-age Z-scores and higher hemoglobin concentrations at 18 months.	Per protocol
FOI-maternal/caregiver delivery → length and hemoglobin concentration	Children of participants who tried and maintained the treatment behaviors will have higher length-for-age Z-scores and higher hemoglobin concentrations at 18 months.	Per protocol

Abbreviations: FOI, fidelity of implementation; IYCF, infant and young child feeding; PIP, Program Impact Pathway; VHW, village health worker; WASH, water, sanitation, and hygiene.

The ITT analyses will examine the impact of the randomized interventions on an intermediate outcome, one at a time, treating that outcome as an endpoint [15, 20, 22], as well as assessing the role of modifying effects on it. For example, the second intermediate domain of the PIP is VHW performance. We hypothesize that a VHW's performance of SHINE tasks (completion of module delivery visits, knowledge transfer) will differ according to their treatment assignment, and that the performance of VHWs assigned to implement both the WASH and IYCF interventions will be lower than that of VHWs assigned to implement only the WASH or IYCF interventions. Further downstream, we hypothesize that for the WASH intervention, mothers in households with greater access to water will practice hand washing with soap to a greater extent than households with less access to water. These analyses exploit the randomized design, and ITT estimates will be estimated as described [31] and will provide estimates of the average effect of the interventions (ie, the ITT effects) on the intermediate outcomes according to our program theory (PIP). Collectively, these analyses will address (1) the extent to which each of the 4 intermediate sequential processes were achieved; and (2) what the modifiers of those processes were, including whether the effects were modified by predetermined characteristics.

The per-protocol analyses go beyond these intermediate outcome ITT estimates to examine movement along the PIP—that is, the linkages from earlier to later steps in the chain including, in particular, the final outcomes. For example, linking VHW performance capacity to actual VHW performance. Per-protocol analyses will also explore the linkages from earlier steps in the chain to the final outcomes, such as linking FOI of VHW delivery and stunting and anemia (to ascertain the effects among those who received the treatment as intended), and linking FOI of maternal/caregiver delivery to stunting and anemia (effects among those who tried and maintained the treatment behaviors). Conditional on having delivered/received the intervention relevant to the participant's treatment arm (as defined by indicators for FOI), we will examine the association between the intervention and the outcome in a later step of the PIP, as well as with the final outcomes of the trial. As with the first set of ITT analyses, potential modifiers at each stage can be assessed using interactions.

Depending on the starting point, the per-protocol analyses will be based on our categorization of FOI into 2 types—VHW and caregiver. In the first of these, FOI of VHW delivery, we classify participants who had at least 10 of the 15 VHW SHINE scheduled visits, starting at 24 weeks of gestation as having high/adequate fidelity. We standardized the number of VHW visits (15 module delivery contacts) across treatments to ensure that the content, rather than the number of contacts, is what differentiates the treatment groups. A visit is therefore defined by having contact at a scheduled behavior change intervention delivery visit. For FOI of caregiver delivery, we will develop separate and combined compliance indices for the WASH and infant feeding behaviors and apply a similar condition of at least two-thirds of the behaviors implemented.

In contrast to the ITT, for these analyses the estimation sample is limited to those following protocol, and for whom effects are hypothesized to be larger. A limitation to this approach is that it no longer fully exploits the randomized design and therefore weakens causal inference. A benefit to this approach, however, is that it allows us to explore more directly the links between improved WASH and infant feeding practices themselves and the final outcomes.

In particular, evidence on the linkages along the intermediate stages of the PIP, as well as any dose-response associations in the relationships between VHW delivery of interventions and the final outcomes, can provide additional plausibility to any observed ITT effects. Furthermore, identifying the drivers of effect heterogeneity can elucidate the circumstances, persons, and contexts in which any such effects are likely to be greatest.

## CONCLUSIONS

There are numerous examples in the literature of large-scale interventions that had disappointing health outcomes [45, 46]. In negative studies without a strong process evaluation, it is difficult to discern whether the result was due to poor alignment of the intervention components with the causes of the problem leading to poor efficacy (theory failure) or due to a failure to implement the intervention as designed (implementation failure). In the SHINE Trial, we measure and report the process evaluation based on the detailed PIP we developed to augment the probability design with plausibility inferences. Also, by using the PIP for ongoing process evaluation and course correction, we are increasing the likelihood that the trial tests the main underlying theory of interest. This program impact pathway approach is one way of going beyond the intention to treat, to understand more fully the effects of programs and interventions on the treated.

## Supplementary Data

Supplementary materials are available at *Clinical Infectious Diseases* online (http://cid.oxfordjournals.org). Supplementary materials consist of data provided by the author that are published to benefit the reader. The posted materials are not copyedited. The contents of all supplementary data are the sole responsibility of the authors. Questions or messages regarding errors should be addressed to the author.
